# Supplementary material for: Three-dimensional tracking of the ciliate Tetrahymena reveals the mechanism of ciliary stroke-driven helical swimming
Source: Commun Biol. 2021 Oct 21;4:1209. doi: 10.1038/s42003-021-02756-0 (PMC8531007; doi:10.1038/s42003-021-02756-0)
Supplement: Supplementary file 2 — Supplementary Information [file 42003_2021_2756_MOESM2_ESM.pdf]

Supplementary information for

Three-dimensional tracking of the ciliate *Tetrahymena* reveals the mechanism of ciliary stroke-driven helical swimming

Akisato Marumo[1], Masahiko Yamagishi[1], and Junichiro Yajima[1,2,3]*

[1]*Department of Life Sciences, Graduate School of Arts and Sciences, The University of Tokyo, 3-8-1 Komaba, Meguro-ku, Tokyo 153-8902, Japan*

[2]*Komaba Institute for Science, The University of Tokyo, 3-8-1, Komaba, Meguro-ku, Tokyo 153-8902, Japan*

[3]*Research Center for Complex Systems Biology, The University of Tokyo, 3-8-1, Komaba, Meguro-ku, Tokyo 153-8902, Japan*

*Correspondence should be addressed to J.Y. (yajima@bio.c.u-tokyo.ac.jp)

Supplementary Figures

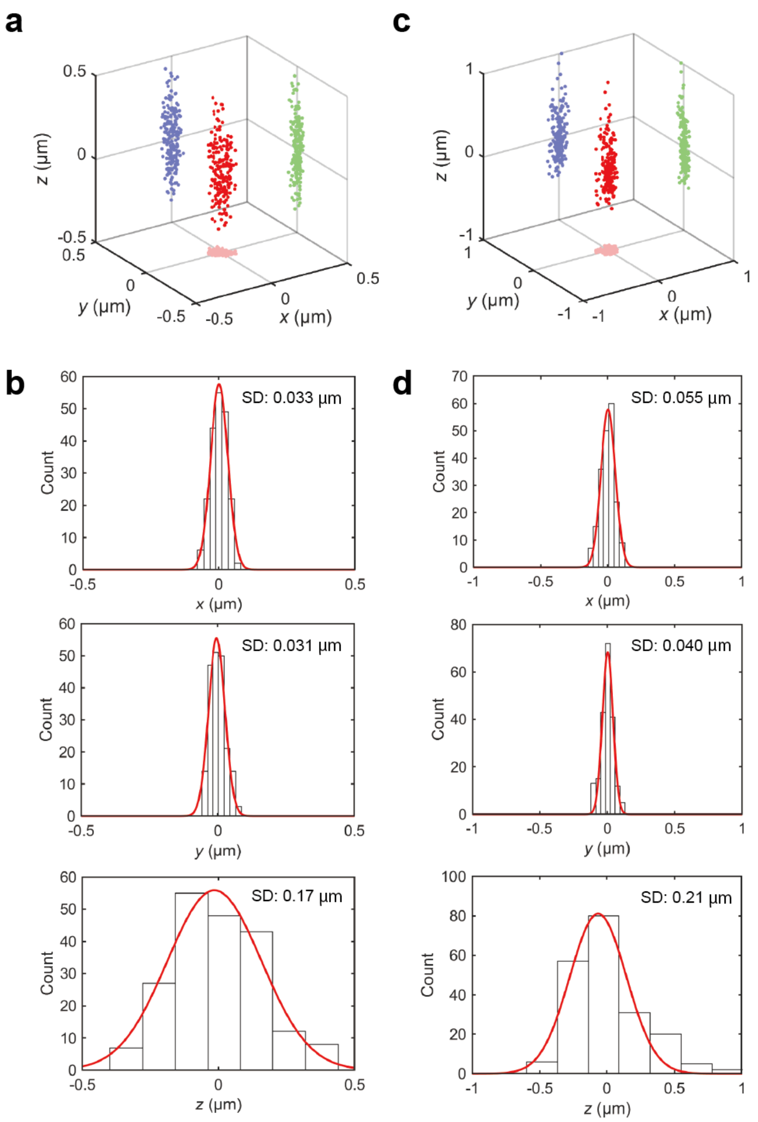

Supplementary Figure 1 | Tracking uncertainty of 3D measurement using the *tPOT* microscope. a, c 3D plots show the positions of a 0.2-μm (diameter) bead fixed in the agarose gel in the observation chamber using a 10× objective (**a**) and a 4× objective (**c**) during 2.2-s recording at 11.21 ms per frame. 3D (red), *x*–*y* (pink), *x*–*z* (blue), and *y*–*z* (green) plots are shown. **b, d** Histograms of *x*, *y*, and *z* positions of the 0.2-μm bead shown in (**a**) and (**c**), respectively. The standard deviations (SD) were derived from a Gaussian fitting.

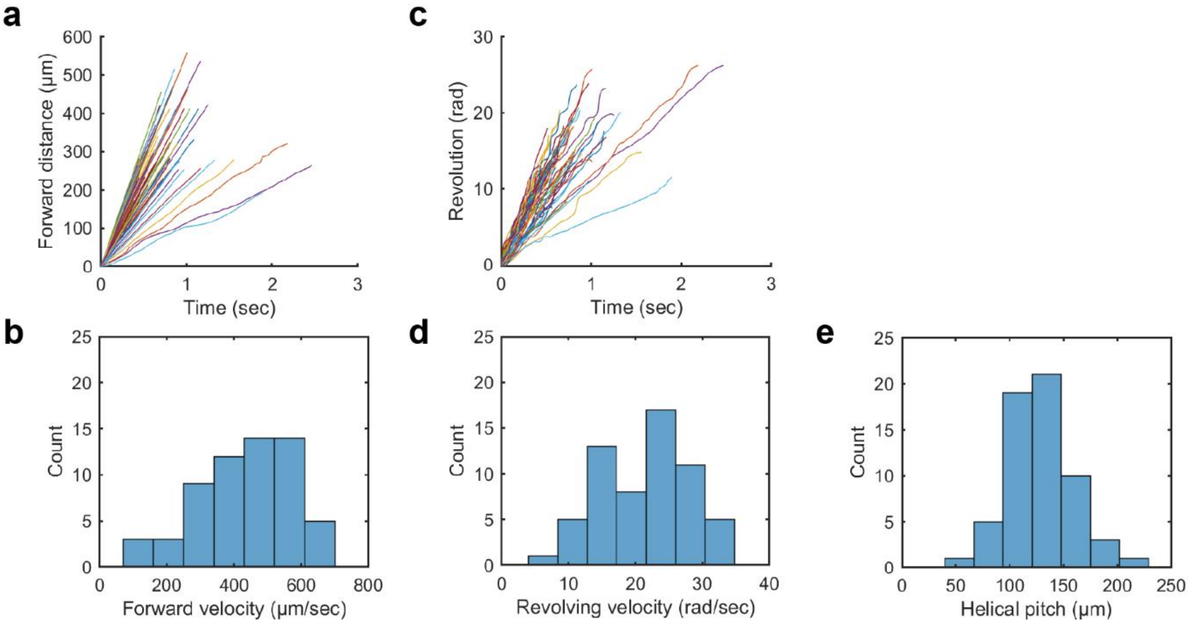

Supplementary Figure 2 | Swimming parameters of *T. thermophila*. **a** Time course of forward distance of beads in swimming *T. thermophila*. **b** Histogram of the forward velocity. Individual traces in **a** were fitted with linear functions to obtain the forward velocity. Average forward velocity: 435 ± 138 µm s^{-1} (mean ± SD, n = 60 cells). **c** Time course of revolution of beads in swimming *T. thermophila*. **d** Histogram of the revolving velocity. Individual traces in **c** were fitted with linear functions to obtain the revolving velocity. Average revolving velocity: 21.4 ± 6.5 rad s^{-1} (mean ± SD). **e** Histogram of the helical pitches of helical trajectories. Individual data in **b** were divided by individual data in **d** to obtain the helical pitch. Average helical pitch: 129 ± 29 µm (mean ± SD). Assays were carried out at 23 ± 1 °C.

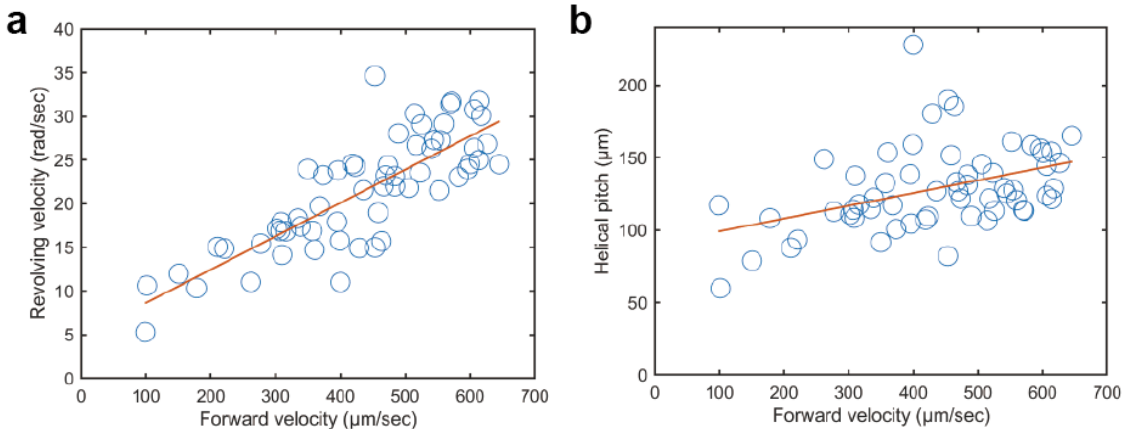

Supplementary Figure 3 | Correlation of revolving velocity and helical pitch with forward velocity of *T. thermophila*. a Forward velocity and revolving velocity (correlation coefficient $R = 0.81$, $n = 60$ cells). **b** Forward velocity and helical pitch ($R = 0.42$). A four-fold decrease in forward swimming velocity, with a subsequent reduction in revolving velocity, is shown. However, there was only a little effect on the helical pitch.

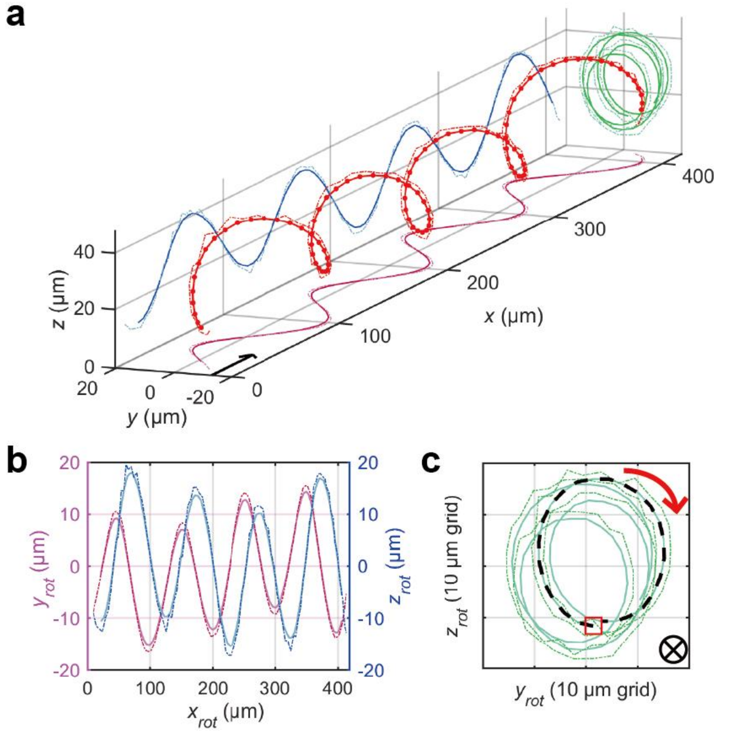

Supplementary Figure 4 | Three-dimensional swimming trajectory of *T. pyriformis*. a 3D plot of the bead (red) in a cell revealed right-handed helical swimming of *T. pyriformis*. The dotted lines show the data acquired at 89 frames s^{-1}, and the solid lines show the data averaged over every 6 frames. Arrow indicates the direction and distance of swimming over 100 ms along the *x*-axis. **b, c** The *x*–*y* (pink) and *x*–*z* (blue) trajectories (**b**) and the *y*–*z* trajectory (**c**) of the bead in the cell in (**a**). Axes are rotated so that *x*-axis is parallel to the swimming direction. Trajectory of the first revolution is shown by the dotted black line and begins at the red open square (**c**). Based on this analysis, the handedness (red arrow) was checked. In this case, the swimming parameters, namely forward velocity, revolving velocity, and helical pitch were 384 µm s^{-1}, 24 rad s^{-1}, and 100 µm, respectively.

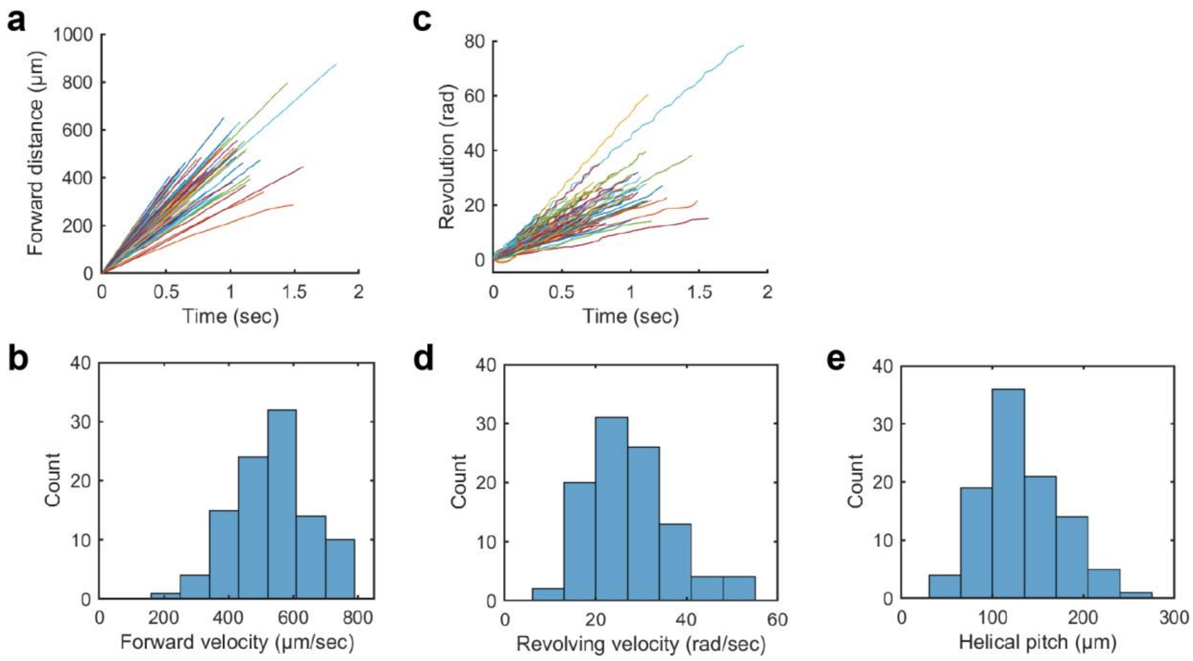

Supplementary Figure 5 | Swimming parameters of *T. pyriformis*. a Time course of forward distance of beads in swimming *T. pyriformis*. **b** Histogram of the forward velocity. Individual traces in **a** were fitted with linear functions to obtain the forward velocity. Average forward velocity: 535 ± 119 μm s^{-1} (mean ± SD, n = 100 cells). **c** Time course of revolution of beads in swimming *T. pyriformis*. **d** Histogram of the revolving velocity. Individual traces in **c** were fitted with linear functions to obtain the revolving velocity. Average revolving velocity: 27.4 ± 8.9 rad s^{-1} (mean ± SD). **e** Histogram of the helical pitches of helical trajectories. Individual data in **b** were divided by individual data in **d** to obtain the helical pitch. Average helical pitch: 132 ± 43 μm (mean ± SD). Assays were carried out at 22 ± 1 °C.

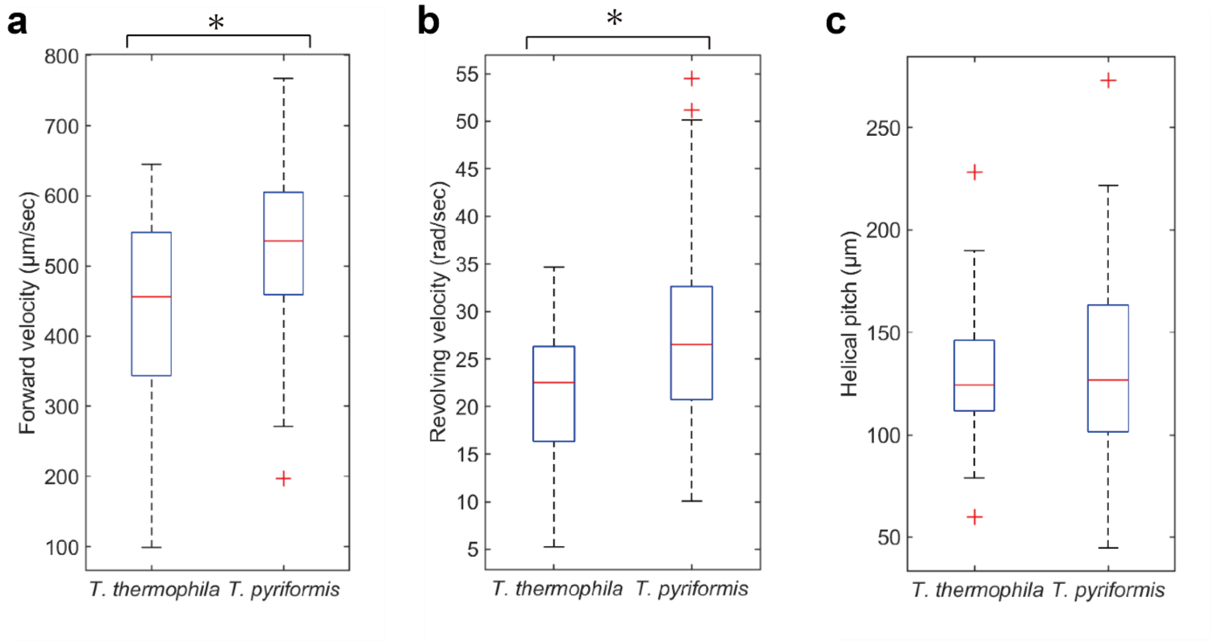

Supplementary Figure 6 | Comparison of swimming parameters between *T. thermophila* and *T. pyriformis*. a Forward velocity of *T. thermophila* (435 ± 138 µm s^{-1} (mean ± SD, n = 60 cells)) was significantly lower than that of *T. pyriformis* (535 ± 119 µm s^{-1} (mean ± SD, n = 100 cells)) ($p < 0.005$, Wilcoxon rank-sum test). **b** Revolving velocity of *T. thermophila* (21.4 ± 6.5 rad s^{-1}, mean ± SD) was significantly lower than that of *T. pyriformis* (27.4 ± 8.9 rad s^{-1}, mean ± SD) ($p < 0.005$, Wilcoxon rank-sum test). **c** Helical pitch did not show such a reduction (*T. thermophila*: 129 ± 29 µm (mean ± SD); *T. pyriformis*: 132 ± 43 µm (mean ± SD)) ($p = 0.79$ with Wilcoxson rank-sum test). In these plots, the red plus sign represents an outlier, defined as a value that is more than 1.5 times the interquartile range away from the bottom or top of the box. See Supplementary Figures 2 and 5 for individual data.

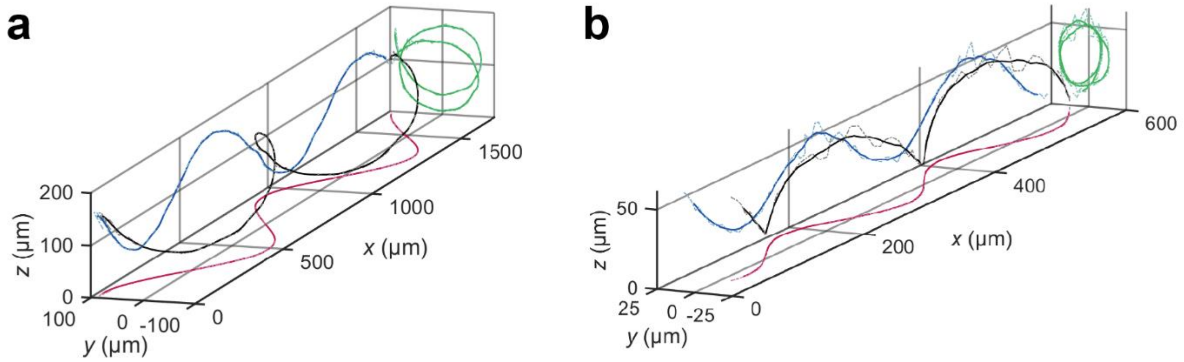

Supplementary Figure 7 | Three-dimensional swimming trajectory of *Paramecium*. a, b An example of the three-dimensional swimming trajectory of *P. multimicronucleatum* (**a**) and *P. calkinsi* (**b**). The shape of these helical trajectories is left-handed (**a**) and right-handed (**b**). The dotted lines show the data acquired at 89 frames s^{-1}, and the solid lines show the data averaged over every 6 frames. In these cases, the forward velocities, revolving velocities, and helical pitches (as swimming parameters) were (**a**) 727 µm s^{-1}, 5.6 rad s^{-1}, and 821 µm, respectively, and (**b**) 531 µm s^{-1}, 14 rad s^{-1}, and 233 µm, respectively. Assays were carried out at 22.5 ± 1.5 °C.

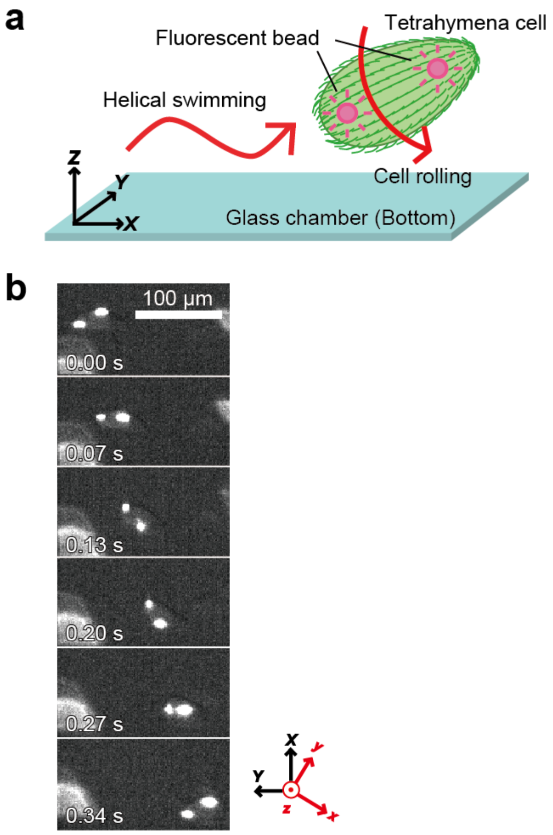

Supplementary Figure 8 | A swimming *T. thermophila* with two separate beads. a Diagram of *Tetrahymena* swimming after ingestion of fluorescent beads (not to scale). Two separate light spots in the same cell body are shown. **b** Sequential images of two separate light spots corresponding to fluorescent beads in separate food vacuoles in a swimming cell, as observed under the *tPOT* microscope. The front of the cell is facing right in this image. This image shows only one side of the field of view divided by the prism in *tPOT* microscope.

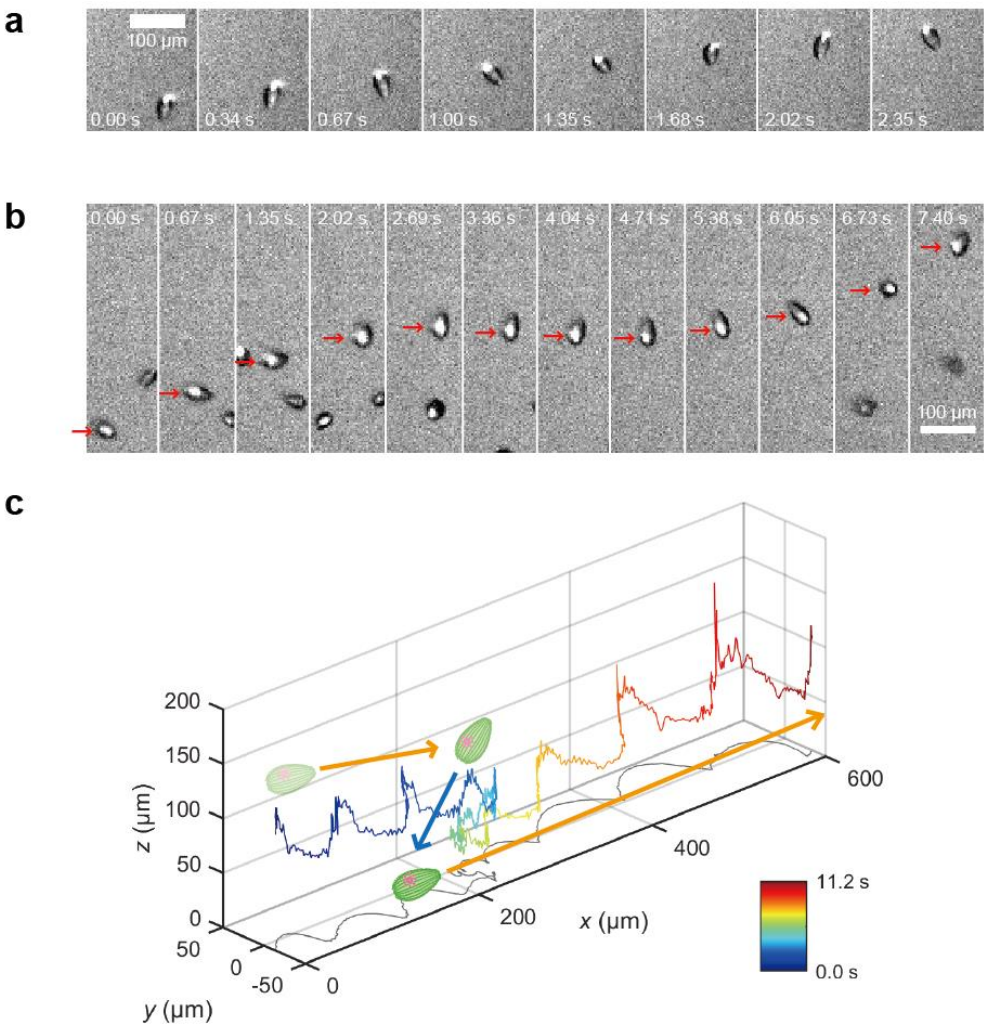

Supplementary Figure 9 | Sequential images of various swimming patterns of *Tetrahymena*. Both cell shape and the fluorescent bead in the cell were observed simultaneously. **a** Sequential images of a backward swimming cell. Front of the cell is facing down in this image. **b** Sequential images of a cell (indicated by the red arrows) swimming forward along a left-handed helical path, backward along a right-handed helical path, and forward along a left-handed helical path, again. Front of the cell is facing up in this image. These images show only one side of the field of view divided by the prism in *tPOT* microscope. **c** An example of a 3D swimming trajectory of *T. thermophila* immediately after depolarizing stimulation with Ca^{2+}. The images represent the orientations of the cell body, and the blue and orange arrows indicate the swimming directions. The cell swam forward along a left-handed helical path, swam backward along a right-handed helical path, and then swam forward along a left-handed helical path, again.

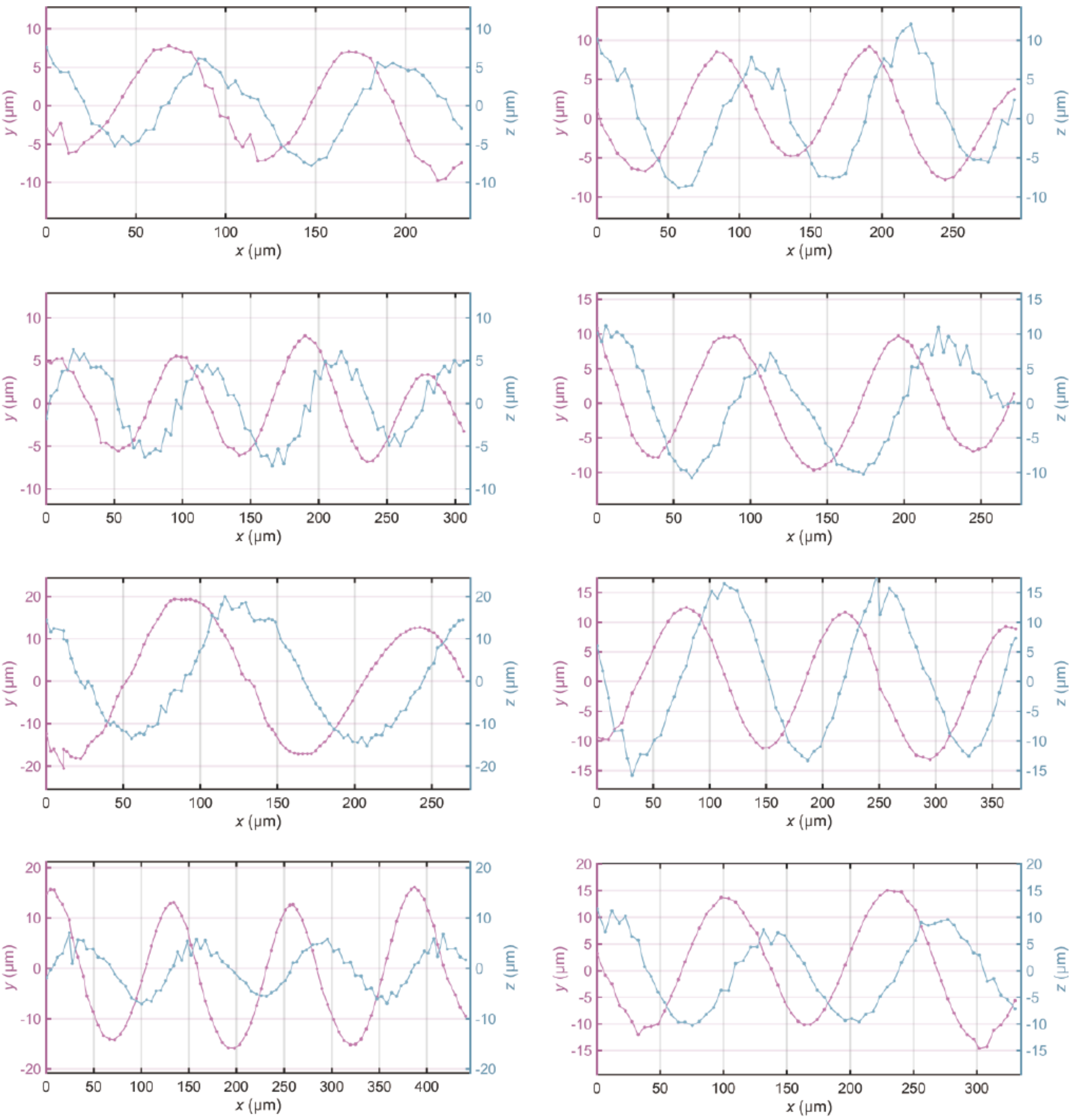

Supplementary Figure 10 | Raw data of *x*–*y* (pink) and *x*–*z* (blue) trajectories of the beads during *T. thermophila* cell swimming in free-space. The trajectories of 8 cells are shown. Data were acquired at 89 frames s^{-1}. Axes are rotated so that the *x*-axis is parallel to the swimming direction. However, the effect of periodic ciliary beating was not detected by these trajectories.
