# Supplementary material for: Three-dimensional tracking of the ciliate Tetrahymena reveals the mechanism of ciliary stroke-driven helical swimming
Source: Commun Biol. 2021 Oct 21;4:1209. doi: 10.1038/s42003-021-02756-0 (PMC8531007; doi:10.1038/s42003-021-02756-0)
Supplement: Supplementary file 3 — Description of Additional Supplementary Files [file 42003_2021_2756_MOESM3_ESM.pdf]

Description of Additional Supplementary Files

File name: Supplementary Movie 1
Description: Swimming of T. thermophila recorded by only one focal plane observation using normal optical microscope. This movie shows the trajectory of the swimming cell, which appears to different observers to be following either a right- or left-handed helical path due to an optical illusion, for ~7.8 seconds (22 ms intervals, ×0.5 actual speed). The image is 234 μm high and 277 μm wide.

File name: Supplementary Movie 2
Description: Swimming of T. thermophila which intakes the fluorescent bead recorded by tPOT microscope. This movie shows both sides of the field of view split by the prism of tPOT microscope for ~1.3 seconds (11 ms intervals, ×1 actual speed). The image is 362 μm high and 439 μm wide.

File name: Supplementary Movie 3
Description: Swimming of P. multimicronucleatum recorded by only one focal plane observation using normal optical microscope. This movie shows the trajectory of the swimming cell, which appears to different observers to be following either a right- or left-handed helical path due to an optical illusion, for ~5.5 seconds (5.5 ms intervals, ×1 actual speed). The image is 668 μm high and 446 μm wide.

File name: Supplementary Movie 4
Description: Ca2+ stimulated backward swimming of T. thermophila. The cell (indicated by the red circle in the movie) swam backward along right-handed helical path and then forward along right-handed path. This movie shows only one side of the field of view imaged by tPOT microscope for ~12 seconds (11 ms intervals, ×1 actual speed). The image is 689 μm high and 466 μm wide.

File name: Supplementary Movie 5
Description: Ca2+ stimulated swimming pattern switching of T. thermophila. The cell (indicated by the red circle in the movie) swam backward along right-handed helical path, forward along left-handed path, backward again along right-handed helical path, and forward again along left-handed path. This movie shows only one side of the field of view imaged by tPOT microscope for ~13 seconds (11 ms intervals, ×1 actual speed). The image is 769 μm high and 364 μm wide.

File name: Supplementary Data 1
Description: Data used to generate the charts and graphs in the main figures.
